# Genome-wide discovery of G-quadruplex forming sequences and their functional relevance in plants

**DOI:** 10.1038/srep28211

**Published:** 2016-06-21

**Authors:** Rohini Garg, Jyoti Aggarwal, Bijal Thakkar

**Affiliations:** 1 National Institute of Plant Genome Research (NIPGR), Aruna Asaf Ali Marg, New Delhi, India

## Abstract

DNA, in addition to the canonical B-form, can acquire a variety of alternate structures, such as G-quadruplexes. These structures have been implicated in several cellular processes in animals. In this study, we identified different types of G-quadruplex forming sequences (GQSes) in 15 sequenced plants and analyzed their distribution in various genomic features, including gene body, coding, intergenic and promoter regions. G2-type GQSes were most abundant in all the plant species analyzed. A strong association of G3-type GQSes with intergenic, promoter and intronic regions was found. However, G2-type GQSes were enriched in genic, CDS, exonic and untranslated regions. Further, we identified GQSes present in the conserved genes among monocots and dicots. The genes involved in development, cell growth and size, transmembrane transporter, and regulation of gene expression were found to be significantly enriched. In the promoter region, we detected strong co-occurrence of Telobox, ERF, MYB, RAV1B and E2F motifs with GQSes. Further, we validated the structure formation of several plant GQSes, demonstrated their effect on stalling *in-vitro* replication and revealed their interaction with plant nuclear proteins. Our data provide insights into the prevalence of GQSes in plants, establish their association with different genomic features and functional relevance.

DNA can exist in a variety of three-dimensional structures, such as B-form, Z-form and G-quadruplexes, inside a cell^1^,^2^. G-quadruplex is one of the non-canonical four-stranded structure made up of multiple Hoogsteen base-paired G-quartets stacked on top of each other^2^. These have been found to be enriched in functional regions of the genome, such as genes, promoters, telomeres and untranslated regions (UTRs) of mRNA^3^,^4^,^5^,^6^,^7^,^8^,^9^. Induction or stabilization of G-quadruplex in promoters and mRNA has been shown to regulate gene expression and translation, respectively^10^,^11^,^12^,^13^,^14^,^15^. Until recently, formation of these G-quadruplex structures in cells was questionable. However, recent experiments with human cell lines have established the formation of G-quadruplexes in DNA and RNA in eukaryotic cells^16^,^17^,^18^,^19^,^20^.

Various G-quadruplex prediction algorithms, such as QuadParser, QGRS-Conserve, QGRS mapper and G4P Calculator have been developed based on various biophysical studies on *in vitro* G-quadruplex formation by oligonucleotides^21^,^22^,^23^,^24^. G-quadruplex forming sequences (GQSes) have been categorized into different types based on the number of guanine repeats (2-G2, 3-G3 or 4-G4) and number of nucleotides in the loops (loop length of 1–3 bp, 1–7 bp and so on). Stability of G-quadruplex is dependent on many of these factors, such as loop length, number of G-repeats, and cation (K^+^ or Na^+^) availability^23^,^25^. The predicted stability is maximum for shorter loop length as compared to longer loop length. This means GQSes with loop length of 1–3 bp have highest stability followed by GQS with loop length of 4–5 bp or loop of 6–7 bp followed by longer loops, bulges and others^23^. G3-type G-quadruplexes are more stable with loop length of 1–3 bp or 1–7 bp, similarly G2-type G-quadruplexes are more stable with loop length of 1, 1–2 bp, or 1–4 bp^23^.

Considerable advances have been made in understanding the role of G-quadruplexes in regulation of gene expression, maintenance of telomeres and regulation of translation in human and yeast^26^,^27^,^28^,^29^,^30^. GQSes have been identified in human telomeric regions, promoter regions of many oncogenes (like KRAS, RET, VEGF, c-Myc and Bcl-2), and immunoglobulin switch regions^26^,^28^,^31^,^32^,^33^,^34^,^35^. It has been shown that different GQS motifs are enriched in different regions of the genome^5^,^6^,^7^. For example, GQSes present in promoters of MYC and KRAS could regulate their expression^26^,^28^. Similarly, it has been shown that G-quadruplexes formed at the telomere ends are the substrates for telomerase enzyme^30^. Although some reports have identified putative GQSes in few plant species^36^,^37^, a comparative genome-wide analyses is lacking. Further, their role in regulation of various biological processes has also not been explored as of now.

In this study, we identified putative GQSes in various sequenced plant genomes, and studied their genome-wide distribution and association with different genomic features. We identified orthologous genes in monocots and dicots harboring GQSes within gene body or promoter regions. Further, we have revealed the *cis*-regulatory motifs enriched in GQSes present within the promoter sequences. G-quadruplex formation by several of the identified GQSes has been demonstrated and their effect on *in-vitro* DNA replication was established. In addition, we demonstrated the structure-specific binding of GQSes with plant proteins. Our results provide a framework for future studies on various regulatory roles of GQSes in plants.

## Results and Discussion

### Genome-wide discovery of putative GQSes in plants

**Figure 1:**
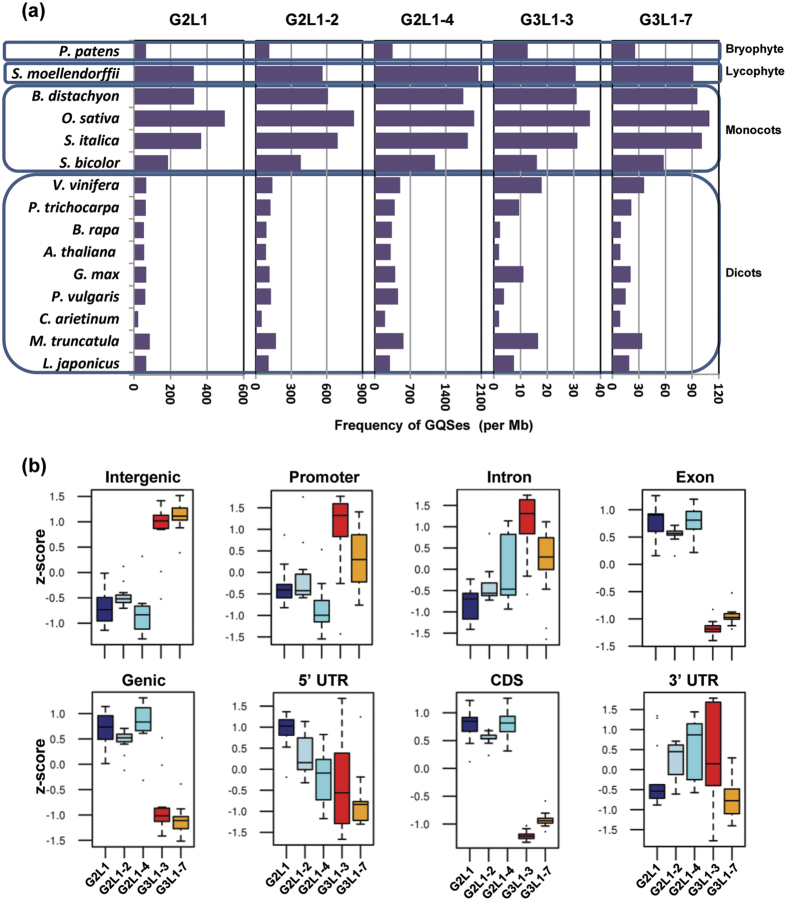
Frequency and distribution of various GQSes in the plant genomes. (**a**) The bar graph displays the frequency (per Mb) of GQS motifs (G3L1-7, G3L1-3, G2L1-4, G2L1-2 and G2L1) in different plant genomes. Blue boxes represent classification of species as depicted on left side. (**b**) Box-plots showing the enrichment of different GQS motifs (G3L1-7, G3L1-3, G2L1-4, G2L1-2 and G2L1) within different genomic features (various gene components and intergenic regions) across all plant species.

The genome sequences of 15 plants, including *Arabidopsis thaliana, Oryza sativa, Glycine max, Cicer arietinum, Medicago truncatula, Lotus japonicus, Phaseolus vulgaris, Brassica rapa, Setaria italica, Brachypodium distachyon, Sorghum bicolor, Populus trichocarpa, Vitis vinifera, Selaginella moellendorffii* and *Physcomitrella patens,* were scanned for the presence of putative GQSes. We searched for two or three G repeats with loop length varying from 1 to 1–3, 1–4 or 1–7 bp (i.e G2L1, G2L1-2, G2L1-4, G3L1-3 and G3L1-7) in the plant genomes. Highest frequency was detected for G2L1-4 type GQSes followed by G2L1-2, G2L1, G3L1-7 and G3L1-3 types across all the plant species analyzed (Table 1). The number of G2L1-4 type GQSes were highest in all the plant species analyzed (ranging from 42220 in *A. thaliana* to 743556 in *S. italica*) (Table 1). G3L1-3 GQSes were represented least in number among all types of GQSes (ranging from 260 in *A. thaliana* to 13437 in *O. sativa*). More than 90% of the GQSes identified were of G2-type, whereas G3-type constituted less than 5% of the total GQSes identified in each of the plant species. The frequency of GQSes varied from ~10 to 20 GQSes/Mb in dicots and ~80 to 1500 GQSes/Mb in monocots (Fig. 1a). GQS density in lycophyte, *S. moellendorfii* (spikemoss), was similar to monocots, whereas in bryophyte, *P. patens* (moss), it was similar to dicots. A higher G2-type GQS frequency was observed as compared to G3-type GQS in these non-vascular plants, similar to that in monocots and dicots. Overall, monocots showed higher frequency of all the GQS types as compared to dicots (Fig. 1a). It may be due to higher GC content in monocots as compared to non-monocot angiosperms, gymnosperms or lycophytes^38^. This supports the observed differences in GQS density between monocots and dicots. These results also suggest the advantage of GC-rich DNA (such as GQS) to undergo conformation changes as compared to GC-poor DNA. These conformational changes might contribute to complex genome regulation processes. It will be interesting to understand the link between GQS formation, nucleotide composition and regulation of gene expression. Although an earlier study reported analyses of GQSes in plants, it was restricted to eight plant species and G3L1-7 type GQS only^36^.

### Distribution of GQSes in various genomic features

An earlier study provided evidence for enrichment of specific type of GQSes with different genomic regions in Arabidopsis^36^. To gain better insights about the specificity of association of different types of GQSes with different genomic regions, we analyzed their distribution in different regions/features of the genome, including genic (exons, introns and UTRs) and intergenic regions and promoters in the 15 plant species (Table S1). The percentage of G2-type GQS in genic regions varied from 9% in *L. japonicus* to 44% in *S. moellendorfii*, while it varied from 55% in *S. moellendorfii* to 90% in *L. japonicus* in intergenic regions. Similarly, the percentage of G3-type GQS in genic region varied from 7% in *S. italica* to 38% in *S. moellendorfii*. However, it varied from 61% in *S. moellendorfii* to 92% in *L. japonicus, S.italica* and *C. arietinum* in intergenic regions (Table S1). Overall, the percentage of all types of GQSes in the intergenic region was higher than percentage of GQSes in the genic region in all the plant species (19.38% of G2/G3 GQSes in the genic region vs 80.61% in the intergenic region). Within the genic region, the percentage of G2-type GQSes was higher (16.9%) in the exonic region as compared to G3-type GQSes (7.7%). In contrast, percentage of G3-type GQSes was higher in the intronic regions (7.2%). The enrichment of various types of GQSes in different regions of the genome in all the plant species was computed. The results indicated significant enrichment of G3-type GQSes (G3L1-3 and G3L1-7) in intergenic (p-value = 4.6e-25), promoter (p-value = 2.21e-7) and intronic regions (p-value = 1.3e-6). However, G2-type GQSes (G2L1, G2L1-2, G2L1-4) were significantly enriched in genic (p-value = 4.6e-25), CDS (p-value = 8.39e-49), and exonic regions (p-value = 2.89e-48) (Fig. 1b). G2L1-4 was found to be enriched in the intronic regions also (p-value = 0.014). G2L1 was enriched in 5′-UTR (p-value = 1.29e-8), and G3L1-3, G2L1-2 and G2L1-4 were found to be enriched in 3′-UTR (p-value = 4.5e-5, 0.04, 0.006). A similar pattern of enrichment of G2- and G3-type GQSes in genic and intergenic regions has been reported earlier in Arabidopsis^36^. Overall, these observations indicated the specificity of association of different types of GQSes with different genomic regions. It appears that G3-type GQSes might have a role in regulating promoter activity, while G2-type GQSes might regulate transcription and translation processes in plants.

### Functional annotation of GQS harboring conserved genes in monocots and dicots

Multispecies comparison is particularly effective for detecting conserved regions and revealing potential common regulatory regions^24^. To investigate the functional relevance of GQSes present in the plant genomes, we analyzed their presence in the genes that are conserved across multiple plant species. We first identified conserved (orthologous) genes within dicots (*A. thaliana, G. max, C. arietinum, M. truncatula, P. vulgaris* and *B. rapa*) and monocots (*O. sativa, S. bicolour, S. italica* and *B. distachyon*). A total of 10080 orthologous genes in dicots and 15903 orthologous genes in monocots were identified. We located the GQSes in the corresponding orthologous genes in *A. thaliana* for dicots and *O. sativa* for monocots. A total of 4569 and 77 orthologous genes were found to harbor G2-type and G3-type GQSes, respectively, within gene body in *A. thaliana*, whereas 1000 and 35 genes harboured G2-type and G3-type GQSes within their 1 kb promoter regions. Similarly, 14634 and 2121 genes of *O. sativa* were identified with G2-type and G3-type GQSes, respectively, within their gene body, whereas 5859 and 639 genes had G2-type and G3-type GQSes, respectively, within their 1 kb promoter. Overall, a higher number of orthologous genes harbouring G2-type GQSes both within gene body and promoter were identified in monocots and dicots (Table S2). Of the 10080 orthologous genes, we identified 1331 orthologous genes harbouring GQSes in all the dicot species, whereas 9786 orthologous genes in the monocots harboured GQSes (Table S3). In addition, 678 orthologous genes were identified to contain GQSes in 1 kb promoter in all the monocot species (Table S2 and S3). The presence of GQSes in similar genomic features of orthologous genes supports association of GQSes with evolutionarily constrained locations relative to gene structures in plants.

**Figure 2:**
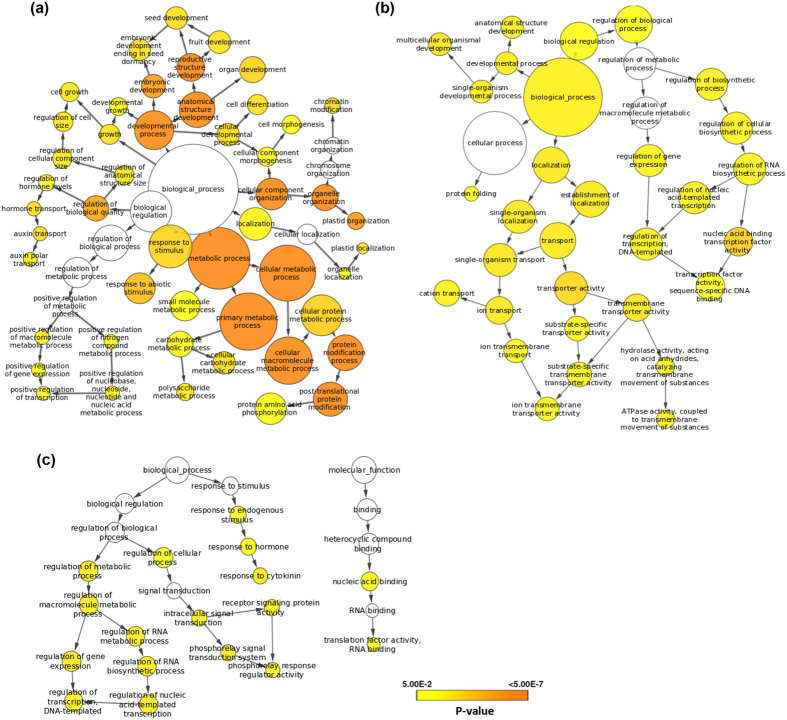
Gene ontology (GO) enrichment of orthologous genes harboring putative GQSes in genic region of all dicot species (**a**) and monocot species (b). The genes were analyzed using BiNGO and the terms showing significant enrichment are shown. Significantly enriched GO categories in genes are shown. Node size is proportional to the number of genes in each category and colors shade represent significance level (white - no significant difference; color scale, yellow - P-value = 0.05, orange - P-value < 0.0000005). (**c**) GO enrichment of orthologous genes harboring GQSes in 1 kb promoter region of monocot species.

**Figure 3:**
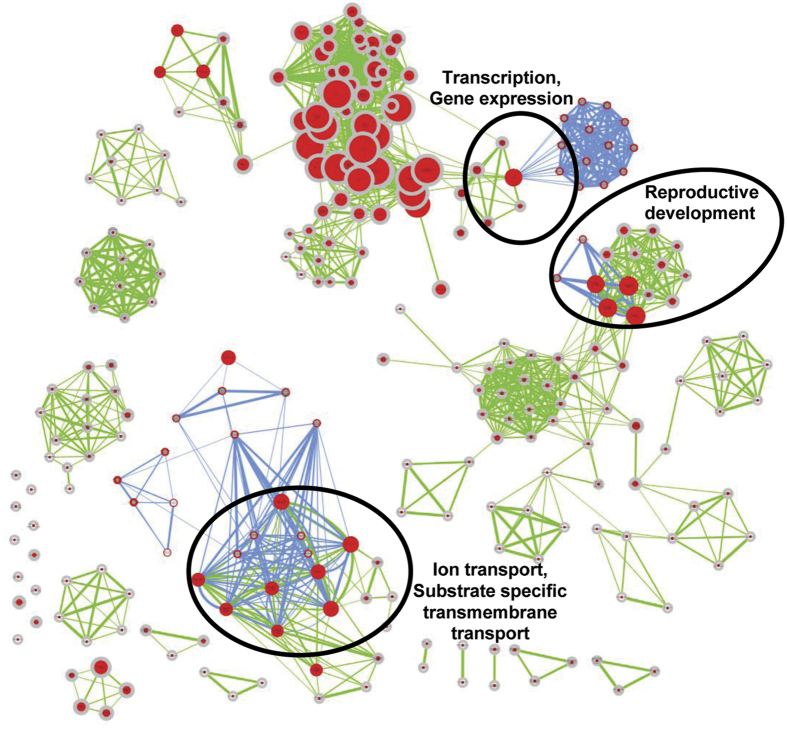
Biological processes conserved among orthologous genes harboring GQSes in monocots and dicots. Orthologous genes harbouring GQSes were analyzed for gene ontology (GO) enrichment using Cytoscape (p ≤ 0.005). Nodes highly enriched in monocot orthologous genes are shown in blue edges while those in dicots are shown in green edges. Node size represents number of genes. Colour of the node and border corresponds to the significance based on the p-value of the gene set. Edge thickness represents the degree of overlap between two gene-sets. Nodes were grouped according to GO definition. The clusters with both green and blue edges are highlighted and annotated with the group names.

**Figure 4:**
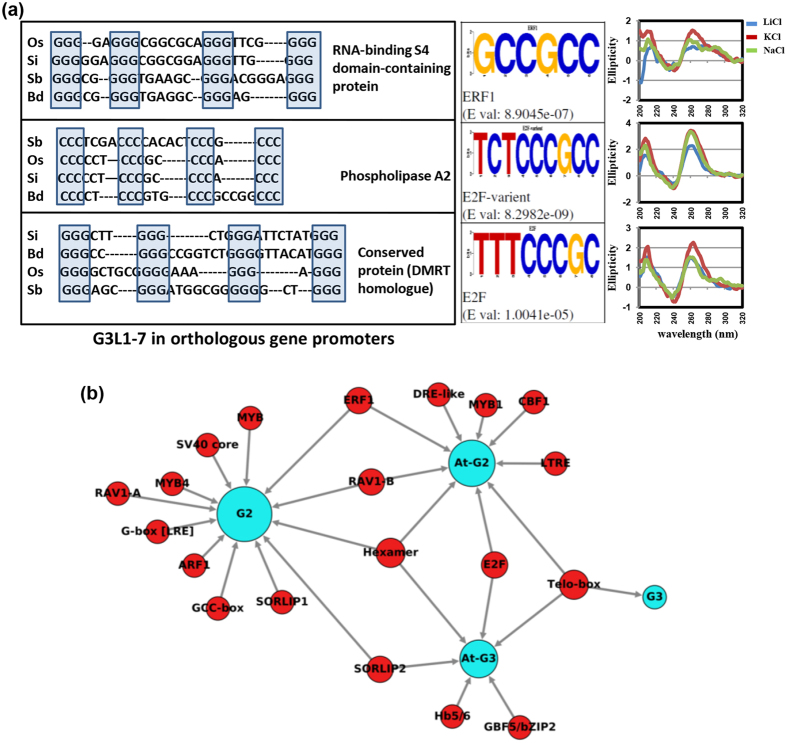
Transcription factor (TF) binding sites identified in GQSes present in promoters of orthologous genes. (**a**) Left panel: G3L1-7 sequences in three promoter associated GQSes of orthologous genes conserved across all monocot species with annotation of rice genes. Os, *Oryza sativa*; Si, *Setaria italica*, Sb, *Sorghum bicolor*, Bd, *Brachypodium distachyon*. Middle panel: Logos showing the TF binding sites in the three OsG3L1-7. Right panel: CD spectroscopy of the rice GQSes. (**b**) TF binding sites (red circles) enriched within 1 kb promoters of Arabidopsis genes harboring GQSes (G3: G3L1-7, G3L1-3; G2: G2L1-4, G2L1-2 and G2L1; blue circles) or within the GQS motifs present in 1 kb promoters in Arabidopsis (AtG2/AtG3; blue circles).

Gene ontology (GO) enrichment analysis revealed that orthologous genes in dicots harbouring GQSes were involved in biological processes, like chromatin modification involving SWI/SNF complex, intracellular signal transduction by regulating phosphorylation, auxin transport and response to gravitropism, pollen morphogenesis, seed development, and GTPase activity etc. (Fig. 2a). Similarly, GQS containing orthologous genes in monocots were also found to be involved in biological processes, such as developmental processes, ion transport, regulation of transcription and protein folding etc. (Fig. 2b). Orthologous genes in monocots harbouring GQSes in the promoter regions were involved in processes, like transcriptional regulation, intracellular signal transduction, response to cytokinin and translational activity (Fig. 2c). In addition, biological processes, such as developmental processes, anatomical structure development, ion transmembrane transport and regulation of gene expression, were common between GQS harbouring orthologous genes in both monocots and dicots (Fig. 3). The conservation of GQSes in the orthologous genes within gene body or promoter in these species suggested their functional implications in the enriched biological processes. For instance, three orthologous rice genes encoding for RNA binding S4 domain protein, phospholipase A2 (PLA2) and a conserved gene of unknown function (expressed highly in reproductive stages) were found to harbour a G3-type GQS in their promoter (Fig. 4a, Table S4). The conserved gene of unknown function (LOC_Os08g05540) is a homologue of doublesex and mab-3 related transcription factor 3 (DMRT) in animals, which is known to be involved in sex determination^39^. Interestingly, this gene lies within the quantitative trait loci (QTL ID: CQE64) controlling tiller number in rice and was found to be highly expressed at pre and post-emergence inflorescence, pistil and seed-5 DAP in rice (as observed in the expression dataset available at Rice Genome Annotation Project webpage). The overlapping expression pattern of this gene in animals and plants suggested its conserved function in reproductive development in plants. The presence of a GQS in its promoter in all the monocots analyzed suggested its possible regulation by G-quadruplex. Similarly, many of the orthologous *Arabidopsis* genes harbouring G3-type GQSes in their promoter were also found to be involved in reproductive processes, such as synergid death and embryo sac central cell differentiation (AT5G48030, encoding for gametophytic factor 2), regulation of pollen tube growth (AT5G12180, encoding for calcium-dependent protein kinase 17), promoter binding (AT2G20570, encoding for Arabidopsis golden2-like 1) and epigenetic regulation of gene expression (AT4G19020, encoding for chromomethylase, CMT2) etc. These results suggest the role of G-quadruplexes in regulating important developmental processes in plants.

### Conserved *cis*-regulatory motifs within GQSes in plants

To further investigate the potential function of GQSes, we analyzed association between transcription factor (TF) binding motifs and GQSes located in the promoter regions. We performed motif search on G3-type GQSes using HOMER followed by STAMP^40^. We found TELO-box, EIN3, SORLIP2, E2F variant, Hexamer and GBF5 (AtbZIP53/bZIP44) motifs enriched in G3-type GQSes. Similarly, we found ERF1, TELO-box, MYB1, DRE-like, DREB1B (CBF1), Hexamer, LTRE, E2F and RAV1-B binding sites to be enriched in G2-type GQSes. Since G3-type GQSes were found to be enriched in promoter sequences, we used all the G3-type GQS containing promoters from *Arabidopsis* and searched for the presence of various motifs^40^. We identified TELO-box as the most significant motif present in the promoters of genes containing G3-type GQSes (Fig. 4b). We also identified SORLIP1/SORLIP2, MYB4/MYB, Hexamer, ERF1/GCC-box, RAV1-B/A, SV40 core, G-box promoter motif [LRE], and ARF1 to be enriched in G2-type GQSes associated promoters in *Arabidopsis* (Fig. 4b). TELO-box has been identified in zones of initiation of DNA replication and flanking regions of several genes^41^. Pura-like DNA-binding protein (Purα) has been identified as interacting partner for TELO-box in *Arabidopsis*^42^. E2F binding sites were found to be enriched in replication origin centers and E2F binding was involved in regulation of cell division^43^. Interestingly, AtPurα was also shown to interact with AtE2F proteins. We also found enrichment of binding site of E2F in GQS present in the promoter of DMRT homologue of plants (Fig. 4a). SORLIP1 and 2 are involved in induction of gene expression under high light^44^. One such example is Early Light-Induced Protein (ELIP) gene promoter, which contains a GQS. LONG HYPOCOTYL5 (HY5), has recently been shown to bind ELIP promoter under high-light and UV-B^45^. Hexamer motif is involved in histone H4 gene expression and meristem development^46^. GCC-box or ERF1-binding motif is present in ethylene and jasmonic acid regulated promoters^47^. RAV1 (EDF1) family members are induced by jasmonic acid and ethylene and involved in leaf senescence^48^. We also found enrichment of GCC box (ERF1 binding sites) in the promoters containing G2-type GQSes. The enrichment of E2F, Hexamer and Telo-box motifs in the GQSes suggest their role in cell-cycle control. Likewise, the enrichment of SORLIP, ERF, MYB and bZIP motifs within GQSes indicates their role in cell growth and development. Overall, the current systematic analysis identified conserved promoter motifs across different plant species. These observations suggest that GQS formation in plants might regulate gene expression by modulating transcription factor binding to specific promoter elements.

### Structural characterization of candidate novel G-quadruplexes in DNA

**Figure 5:**
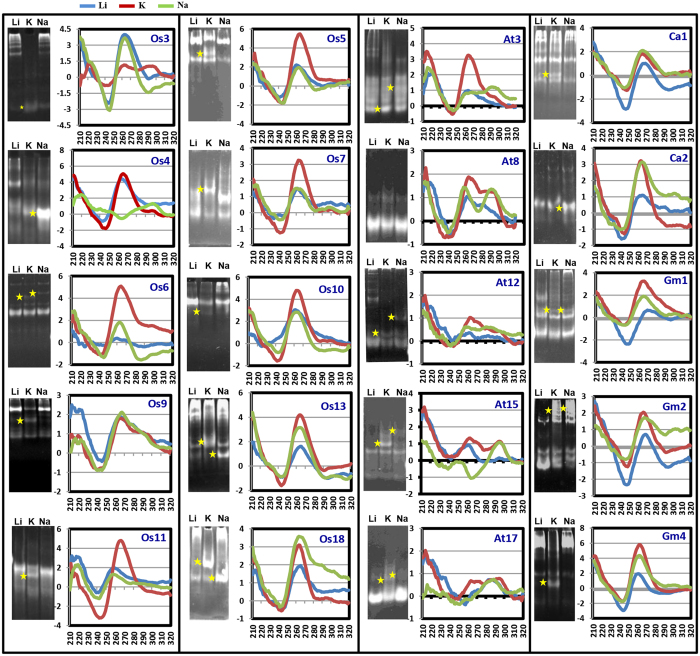
Validation of GQS structure and conformation. Native gel electrophoresis (left panel) and CD spectra (right panel) of selected putative GQS forming oligos in presence of various cations. Li-150 mM LiCl, K-150 mM KCl, Na- 150 mM NaCl. X-axis of CD-spectra depicts wavelength (nm) and Y-axis depicts Ellipticity. Os: *Oryza sativa*; At: *Arabidopsis thaliana*; Gm: *Glycine max*; Ca: *Cicer arietinum*. Oligo ID are given on top right side of CD spectra. All the gels were run under the same experimental conditions and presented by using cropped images. Stars represent shift in mobility of oligos in particular lane.

G-quadruplex structures exhibit characteristic features in circular dichorism (CD)^49^. Thus, CD-spectroscopy along with gel electrophoresis can be used to reveal detailed structural arrangements in G-quadruplexes. To validate the folding of various putative GQSes identified in the above analysis, we performed polyacrylamide gel electrophoresis (PAGE) and CD-spectroscopy experiments. At least, twenty GQSes present in the promoters containing *cis*-regulatory elements (Table S5) were selected from different plants (five from *A. thaliana*, ten from *O. sativa*, three from *G. max* and two from *C. arietinum*) for validation of G-quadruplex structure formation (Table 2). Depending upon the orientation of loops, G-quadruplex can adopt a parallel or antiparallel geometry. A strong positive band at 260 nm in CD spectroscopy implies parallel quadruplexes, whereas a negative band close to 260 nm and positive ones at 295 and 240 nm suggests antiparallel quadruplex. In addition, two positive bands around 260 and 290 nm imply the presence of quadruplex containing three parallel and one antiparallel strand or (3 + 1) hybrid folding topology. We were able to detect parallel (Os6, Os11, Os5, Os7, Os10, Os13, At3, At12, Ca1, Ca2, Gm1, Gm2 and Gm4), antiparallel (Os4, At15 and At17) and hybrid (Os3, Os9, At3, At8, At15, At17, Gm2 and Ca2) G-quadruplex structures in the selected sequences (Table 2 and Fig. 5). However, to ascertain the molecularity (intra or inter- molecular) of GQSes, PAGE was performed. Electrophoretic separation of various folded species shows distinct migration patterns with slowly migrating species corresponding to multimeric intermolecular complexes, while the fastest migrating species are intramolecular^50^. We detected intramolecular G-quadruplex formation for Os5, Os7, At3 and At8, and intermolecular G-quadruplex formation for At12 and Os6 GQSes (Fig. 5 shown by asterisks in gels and Table 2).

The cations (potassium and sodium) have different effects on stability and formation of quadruplex structures^25^. We measured the CD-spectra of each of the above oligos in the presence of K^+^ or Na^+^ ions to assess their effect on G-quadruplex formation (Fig. 5). The CD-spectrum of oligos suggested the formation of parallel G-quadruplex in the presence of K^+^ (Os5, Os6, Os7, Os11, Os10, Os13, At3, At12, Ca1, Ca2, Gm1, Gm2 and Gm4) or antiparallel G-quadruplex in the presence of Na^+^ (Os4, At15 and At17). Similarly, (3 + 1) hybrid folding topology was observed in K^+^ (Os3, At8, At15 and At17) or Na^+^ ions (At3, At8, At12, Ca2 and Gm2). These results demonstrated the ion-dependent formation of G-quadruplex structure with different topologies and molecularity.

### DNA polymerase stalling by plant G-quadruplexes

**Figure 6:**
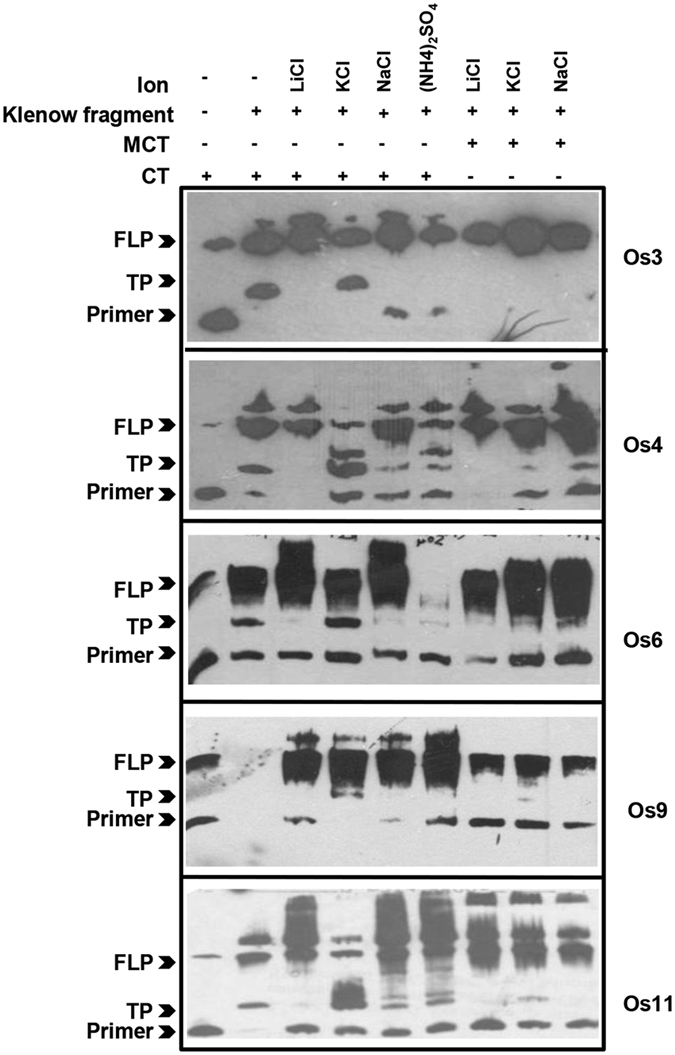
DNA polymerase stalling by GQS formation. Taq polymerase stalling assay in presence of LiCl, NaCl, KCl or (NH_4_)_2_SO_4_ with control (CT) vs mutated (MCT) oligos using biotinylated primers (Primer). Amplified products were loaded on 15% denaturating urea-PAGE transferred to biodyne B membrane and blotted with avidin-HRP. Arrowheads indicate full-length product, FLP; truncated products, TP and biotinylated-primer, Primer. All the gels were run under the same experimental conditions and blots are presented by using cropped images.

Recent evidences indicate a vital role of G-quadruplexes in regulation of DNA replication and transcription^7^,^8^,^9^,^10^,^11^,^12^,^27^,^28^,^51^,^52^. To assess the effect of plant GQSes on DNA replication, we selected several GQSes that can fold in parallel and/or anti-parallel structures and assayed their effect on DNA polymerase activity (Table 3). The oligos containing the control (CT) or mutated (MCT) GQSes were annealed with labeled primer and extended using DNA polymerase. In absence of any secondary structure (LiCl), primer extension occurs till the 3′-end of the oligo. However, G-quadruplex structure formation (in K^+^ or Na^+^), results in a truncated product. We observed that all the sequences that could fold into G-quadruplex structures, were able to inhibit DNA polymerase activity in the presence of K^+^ ions (CT-Os3, Os4, Os6, Os9 and Os11) or Na^+^ (CT-Os4, Os9 and Os11) (Fig. 6). These results were consistent with the formation of G-quadruplex structures in the presence of respective ions (Fig. 5). Further, this ability was specific to G-quadruplex structure formation, because mutation in the sequences (MCT) that abrogates G-quadruplex formation relieved inhibition on polymerase activity (Fig. 6). For example, truncated products were seen with CT-Os3 in K^+^ ions (that forms hybrid G-quadruplex structure), but not with MCT-Os3 in K^+^. Interestingly, we could detect truncated products with mutated Os4 also. Bioinformatics analysis of MCT-Os4 suggested that even the mutated Os4 sequence has the potential to form G2-type G-quadruplex structure, and hence this might be the reason for DNA polymerase stalling with MCT-Os4 in K^+^. Altogether, these results established that formation of G-quadruplex structures can stall DNA polymerase *in vitro*. It would be interesting to study the effect of these GQS in controlling replication in plants.

### Plant nuclear proteins interact with G-quadruplexes

**Figure 7:**
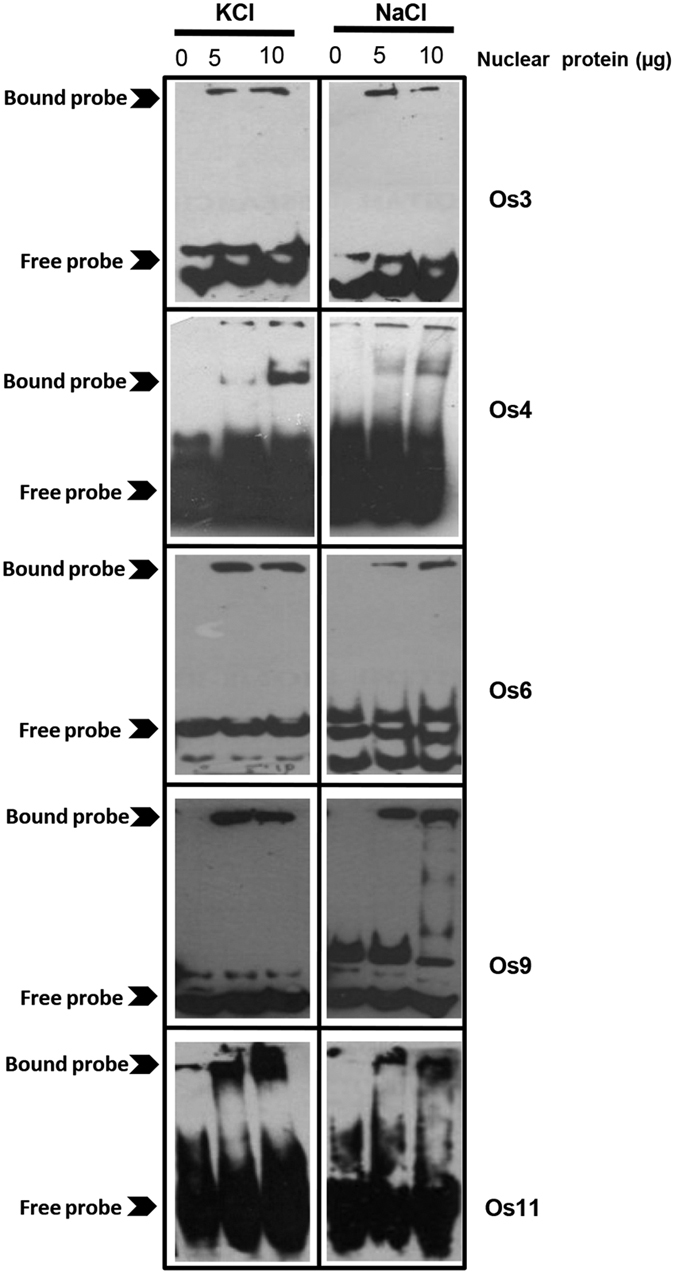
Binding of GQSes to plant nuclear proteins. Electrophoretic mobility shift assay was performed with biotinylated oligos forming G-quadruplex structures and rice nuclear proteins (5 and 10 μg) in the presence of different ions (NaCl and KCl). Protein-DNA complexes were resolved on 6% native acrylamide gel, transferred to biodyne B membrane and blotted with avidin-HRP. All the gels were run under the same experimental conditions and blots are presented by using cropped images.

There have been evidences for binding of proteins to G-quadruplexes in human and yeast^53^,^54^,^55^,^56^. Many of the G-quadruplex interacting proteins are involved in transcriptional regulation (such as PARP1 or mutant p53 proteins) or DNA repair (such as BLM, VRN, XPB and Pif helicases)^53^. However, no evidence of G-quadruplex binding proteins in plants is available till now. To identify plant proteins interacting with G-quadruplexes, we performed electrophoretic mobility shift assay (EMSA) with nuclear extracts from rice plants^54^. Five GQSes identified via bioinformatics approaches followed by validation via SDS-PAGE and CD-spectroscopy experiments, were used to perform EMSA. DNA-protein binding was performed in K^+^ and Na^+^ to identify structure specific protein binding. We were able to detect mobility shift with both parallel (Os3, 6, 9 and 11) and antiparallel (Os4) G-quadruplex structures with rice nuclear protein extracts (Fig. 7). However, the pattern of mobility shift was different in K^+^ and Na^+^ ions consistent with formation of different types of structures in the presence of these ions. This ability to form different DNA-protein complexes with the same sequence suggests that G-quadruplex formation might affect binding of the proteins. Altogether, these results established that formation of G-quadruplex can modulate DNA-protein interactions in plants by promoting/inhibiting binding of proteins to DNA. It would be interesting to study binding of different transcription factors with G-quadruplexes and resultant effect of this interaction in controlling transcription in plants.

## Conclusions

In this study, we identified different types of GQSes in various plant species and reported their genomic distribution. Our analysis showed enrichment of G3-type GQSes in the promoter and non-genic regions, and G2-type GQSes within genic regions of plant genomes. GQSes present in the conserved genes within monocot and dicot species involved in diverse biological processes, were identified. The enrichment of various TF binding motifs within GQSes implied that G-quadruplex formation might regulate binding of these transcription factors to the target promoters. We have provided evidence for adoption of quadruplex structures and their capabilities to inhibit DNA polymerase movement during *in-vitro* replication. Further, we demonstrated the structure-specific binding of GQSes with plant nuclear proteins. The data and result presented here provide framework for studying various regulatory aspects of G-quadruplexes in plants and identification of G-quadruplex binding proteins from plants.

## Methods

### Identification of GQSes

Whole genome sequences of 15 plant species (*A. thaliana, B. rapa, G. max, M. truncatula, L. japonicas, P. vulgaris, C. arietinum, O. sativa, S. bicolour, S. italica, B. distachyon, P. patens, S. moellendorffii, V. vinifera and P. trichocarpa*) were downloaded from Phytozome or their respective genome project databases (Table S6). The genome sequences were scanned using Quadparser tool^5^ for GxNy1GxNy2GxNy3Gx, where x = G2 or G3; y = 1/1–2/1–4 for G2 and 1–3/1–7 for G3. The different categories were defined as follows: loop 1–3, (G3N{1–3})_3_G3 with N = [ATCG]; loop 1–7, (G3N{1–7})_3_G3 and loop 1, (G2N{1})_3_G2, loop 1–2 (G2N{1–2})_3_G2 and loop 1–4, (G2N{1–4})_3_G2. The identified G2-type and G3-type GQSes were mapped on to the gff annotation files of the respective organisms for finding their presence in various genomic features using custom scripts.

### Identification of conserved genes and annotation of GQSes

Protein sequences of *A. thaliana, B. rapa, G. max, M. truncatula, P. vulgaris, C. arietinum, O. sativa, S. bicolour, S. italic* and *B. distachyon* were aligned by reciprocal blast (e-value ≤ 1e-10) and a set of orthologous genes in monocots and dicots were identified. Subsequently, GQSes from each species were mapped on the conserved orthologous genes. GO enrichment of these genes was done via Cytoscape using plug-in BINGO followed by Enrichment map generation^57^.

### Identification of TF binding motifs within GQSes

For identification of TF binding site motifs, we used G2L1-4 and G3L1-7 type GQSes identified within promoter sequences from 10 plants (*A. thaliana, B. rapa, G. max, M. truncatula, P. vulgaris, C. arietinum, O. sativa, S. bicolor, S. italica* and *B. distachyon*). *De novo* motifs of 12 bp length were identified in these GQSes using HOMER2 with their scrambled sequences as background^58^. The motif matrices generated by HOMER were scanned against AGRIS, Athamap and PLACE in STAMP to identify similarity to known TF-binding sites. Similarly, TF binding sites were identified within G2L1-4 and G3L1-7 GQSes identified in promoter region of Arabidopsis genes. Alternatively, we also predicted TF binding sites in promoters of Arabidopsis genes harboring G2L1-4 and G3L1-7 GQSes using elefinder script (http://stan.cropsci.uiuc.edu/cgi-bin/elefinder/compare.cgi) to predict co-occurrence of GQS with TF binding sites.

### Validation of G-quadruplex structure formation

The selected DNA oligos were synthesized commercially (Sigma). The oligos were dissolved in 1x TE buffer and stored overnight at 4 °C. Next day, oligos were denatured at 65 °C for 10 min. An aliquot of 100 μM stock was heated at 95 °C for 15 min, and LiCl/KCl/NaCl was added to a final concentration of 150 mM. Oligos in LiCl were stored at −20 °C, and oligos in KCl and NaCl were kept in the heating block (switched off) for 5–6 h till the temperature of the heating block reached to room temperature. For structure detection, oligos were resolved on 18% acrylamide gel at 50 V, 4 °C. The gel was stained with Gel Red stain (3X). For CD-spectroscopy, 5 μM oligos in 50 mM Tris-Cl (pH 7.5) containing 150 mM LiCl/KCl/NaCl were scanned at wavelength range of 200–320 nm, with 1 nm bandwidth, response time of 0.6 s and path length of 1 mm on Chirascan Spectropolarimeter (Applied Photophysics). Data was buffer subtracted, normalized to provide molar residue ellipticity values and smoothed. Three to five scans of each oligo were averaged.

### EMSA for identification of G-quadruplexes binding proteins

Plant nuclear protein extraction was done using CelLytic PN Isolation/Extraction Kit (Sigma) according to manufacturer’s instructions. EMSA was performed using the LightShift Chemiluminescent EMSA Kit (Pierce). Briefly, 1 μM of biotinylated GQS oligos were incubated with 0, 5 and 10 μg of the nuclear protein in DNA binding buffer (Tris-Cl (pH- 8.0)–10 mM, EDTA–0.5 mM, DTT–0.5 mM, MgCl_2_–1 mM, KCl–50 mM, protease inhibitor -1X and phosphatase inhibitor -1X) along with 1 μl of Poly dI/dC (stock 1 μg/μl) and incubated at 4 °C for 30 min at 30 rpm. The samples were loaded on 6% polyacrylamide gel and resolved in 0.5 X TBE at 20 mA/gel at 4 °C. The samples were transferred to biodyne B nylon membrane in 0.5 X TBE for 90 min at 4 °C, at 380 mA. The membrane was crosslinked in UV cross linker at 254 nM, 120 mJ/cm^2^ for 1 min two times. The blot was subsequently blocked and incubated with HRP-avidin conjugate (1: 5000) and developed using luminal solution provided in the kit.

### Primer extension

12 nM of DNA template (control, CT or mutated, MCT; Table 3) and 12 nM of biotinylated primer were annealed in a reaction of 5 μl in the presence of 150 mM of LiCl/KCl/NaCl/(NH_4_)_2_SO_4_. Subsequently, 2.5 U of Klenow fragment (3′ to 5′ exo-), 0.1 mM dNTP, 1X NEB Buffer, 10 mM DTT, 0.025 μg/μl of Poly dI/dC was added to added to 5 μl of annealed CT/MCT oligo and primer. The reaction was incubated at 37 °C for 30 min and stopped by adding 5 μl of stop buffer (95% formamide, 10 mM EDTA, 10 mM NaOH, 0.1% xylene cyanole, 0.1% bromophenol blue) followed by heating at 70 °C for 5 min. After adding 2 μl of 50% glycerol, samples were immediately transferred on ice. Samples were then resolved on 15% denaturating PAGE containing 8 M urea (gel was pre run for 1 h, in 1 X TBE), in 1 X TBE at room temperature. For biotin labeled primers, samples were transferred to nylon membrane and processed as mentioned above.

## Additional Information

**How to cite this article**: Garg, R. *et al.* Genome-wide discovery of G-quadruplex forming sequences and their functional relevance in plants. *Sci. Rep.* **6**, 28211; doi: 10.1038/srep28211 (2016).

## Supplementary Material

Supplementary Information

Supplementary Dataset

## Figures and Tables

**Table 1:** Number of putative G-quadruplex motifs identified in selected plant species.

Plant species	G2L1	G2L1-2	G2L1-4	G3L1-3	G3L1-7
*Lotus japonicus*	31138	50368	140903	3524	8906
*Medicago truncatula*	20755	40814	135897	3990	8106
*Phaseolus vulgaris*	31856	66476	240084	1985	7892
*Cicer arietinum*	11487	25564	105861	996	4556
*Glycine max*	64877	112705	390940	10761	20189
*Arabidopsis thaliana*	7518	11661	42220	260	1219
*Brassica rapa*	15310	25512	95785	658	2785
*Sorghum bicolor*	134993	275171	862476	11709	42066
*Setaria italica*	149073	280208	743556	12698	40937
*Oryza sativa*	185375	309779	730718	13437	40759
*Brachypodium distachyon*	89189	165547	475214	8437	26044
*Physcomitrella patens*	31223	54234	166629	5997	12138
*Selaginella moellendorffii*	35998	61982	224849	3381	10019
*Vitis vinifera*	32735	67757	243632	8722	17444
*Poplus trichocarpa*	27334	52095	167013	4001	9025

**Table 2:** Oligo sequences used for GQS validation.

Oligo ID	Sequence (5′-3′)	Molecularity	Structural characteristics
Os3	GGGAAGAGGGAAGAGGGGAAGAGGGG	Intramolecular (KCl)	(3+1) and parallel (KCl): Positive peak at 290 and 260 nm
Os4	GGGGGTTGGGGGAGGGTGGGGAAAGTCGGGG	Intramolecular (NaCl)	Anti-parallel (NaCl): Positive peak at 295 nm and negative peak 260 nm
Os6	GGGAGGAGGGAGAAGGGTGGG	Intermolecular (KCl, NaCl): bimolecular and tetramolecular)	Parallel (NaCl): Positive peak at 260 nm (3+1) and parallel (KCl): Positive peak at 290 and 260 nm
Os9	GGGCGCGAGGGAGGAGGGCGCGGG	Intramolecular (KCl, NaCl)	(3+1) and parallel (KCl, NaCl): Positive peak at 290 and 260 nm
Os11	GGGACACGGGGGAGAACTTGGGCATGGGGAGGGTGGGCAGGG	Intramolecular (KCl)	Parallel (KCl): Positive peak at 260 nm
Os5	GGGTCCTAGGGGTGGGGTGGGAAGGGTGGGAGGGGAAGGGGGAGGAGGGA	Intramolecular (KCl)	Parallel (KCl): Positive peak at 260 nm
Os7	GGGTGTGGGGAGGGTGGGG	Intramolecular (KCl)	Parallel (KCl): Positive peak at 260 nm
Os10	GGGGAGGAGGGAGGGTGGGTAGGGGGGGGAGGG	Intramolecular (KCl)	Parallel (KCl): Positive peak at 260 nm
Os13	GGGGAGAGGGAGAAGGGGGAGGAGAAGGGAGAAGGGAGGG	Intramolecular (KCl, NaCl)	Parallel (KCl): Positive peak at 260 nm
Os18	GGGGGGAGGGTGGGGAGTAGGG	Intramolecular (KCl, NaCl)	Parallel (KCl): Positive peak at 260 nm (3+1) and parallel (NaCl): Positive peak at 290 and 260 nm
At3	GGGTGGCGGGAAAATTGGGGACTTAGGG	Intramolecular (KCl)	Parallel (KCl): Positive peak at 260 nm (3+1) and parallel (NaCl): Positive peak at 290 and 260 nm
At8	GGGACGGGTTTGGCGGGACGGG	Intramolecular (NaCl)	(3+1) and parallel (NaCl): Positive peak at 290 and 260 nm
At12	GGGTTGGTTGGATGG	Intermolecular (NaCl)	(3+1) and parallel (KCl, NaCl): Positive peak at 290 and 260 nm
At15	GGTTTGGTTAGGGAGGG	Intramolecular (KCl) Intermolecular (NaCl)	(3+1) and parallel (KCl): Positive peak at 290 and 260 nm Anti-parallel (NaCl): Positive peak at 295 nm and negative peak 260 nm
At17	GGTGGCGTGGCGG	Intramolecular (KCl) Intermolecular (NaCl)	(3+1) and parallel (KCl): Positive peak at 290 and 260 nm Anti-parallel (NaCl): Positive peak at 295 nm and negative peak 260 nm
Ca1	GGGAGAAGGGAGAAGGGAGAAGGGAGAAGGG	Intramolecular (KCl, NaCl)	Parallel (KCl, NaCl): Positive peak at 260 nm
Ca2	GGGGTGGGTGGGTAAGGTGGGG	Intermolecular (KCl, NaCl)	Parallel (KCl): Positive peak at 260 nm (3+1) and parallel (NaCl): Positive peak at 290 and 260 nm
Gm1	GGGAGAAGGGAGAAGGGATGGGGTGGG	Intramolecular (KCl, NaCl)	Parallel (KCl, NaCl): Positive peak at 260 nm
Gm2	GGGAGAAGGGAGAAGGGAAGGG	Intermolecular (KCl, NaCl)	Parallel (KCl): Positive peak at 260 nm (3+1) and parallel (NaCl): Positive peak at 290 and 260 nm
Gm4	GGGTGGGGTTGGGAAGGTGGGAGGAGAGGGTGAGGG	Intramolecular (KCl) Intermolecular (NaCl)	Parallel (KCl): Positive peak at 260 nm Parallel (NaCl): Positive peak at 260 nm

Os: *Oryza sativa*; At: *Arabidopsis thaliana*; Gm: *Glycine max*; Ca: *Cicer arietinum.*

**Table 3:** Oligo sequences containing GQSes used for polymerase stalling.

Oligo ID	Sequence (5′-3′)
Os3_CT	TCCAACTATGTATACTGAAGGGGAAGA**GGGAAGAGGGGAAGAGGGG**AAAATTAGCAACACGCAATTGCTATAGTGAGTCGTATTA
Os3_MCT	TCCAACTATGTATACTGAAG**GGGAAGAGAGAAGAGGAGAAGAGAGG**AAAATTAGCAACACGCAATTGCTATAGTGAGTCGTATTA
Os4_CT	TCCAACTATGTATACTGAAG**GGGGGTTGGGGGAGGGTGGGGAAAGTCGGGG**AAAATTAGCAACACGCAATTGCTATAGTGAGTCGTATTA
Os4_MCT	TCCAACTATGTATACTGAAG**GGGGGTTGGAGGAGAGTGGAGAAAGTCGGGG**AAAATTAGCAACACGCAATTGCTATAGTGAGTCGTATTA
Os6_CT	TCCAACTATGTATACTGAAG**GGGAGGAGGGAGAAGGGTGGG**AAAATTAGCAACACGCAATTGCTATAGTGAGTCGTATTA
Os6_MCT	TCCAACTATGTATACTGAAG**GAGAGGAGAGAGAAGAGTGAG**AAAATTAGCAACACGCAATTGCTATAGTGAGTCGTATTA
Os9_CT	TCCAACTATGTATACTGAAG**GGGCGCGAGGGAGGAGGGCGCGGG**AAAATTAGCAACACGCAATTGCTATAGTGAGTCGTATTA
Os9_MCT	TCCAACTATGTATACTGAAG**GGACGCGAGAGAGGAGAGCGCGAG**AAAATTAGCAACACGCAATTGCTATAGTGAGTCGTATTA
Os11_CT	TCCAACTATGTATACTGAAG**GGGACACGGGGGAGAACTTGGGCATGGGGAGGGTGGGCAGGG**AAAATTAGCAACACGCAATTGCTATAGTGAGTCGTATTA
Os11_MCT	TCCAACTATGTATACTGAAG**GGGACACGGAGGAGAACTTGAGCATGGAGAGAGTGGGCAGGG**AAAATTAGCAACACGCAATTGCTATAGTGAGTCGTATTA
Bio_Primer	[Biotin]TAATACGACTCACTATAGCAATTGCGTG

Sequences in bold and underline represent G residues predicted to be involved in GQS formation and their mutated counterparts in MCT oligos. CT: control oligos; MCT: mutated oligos.
