# Supplementary material for: Genome-wide discovery of G-quadruplex forming sequences and their functional relevance in plants
Source: Sci Rep. 2016 Jun 21;6:28211. doi: 10.1038/srep28211 (PMC4914980; doi:10.1038/srep28211)
Supplement: Supplementary Information [file srep28211-s1.pdf]

*Correspondence and requests for materials should be addressed to

R.G. (rohini@nipgr.ac.in)

Genome-wide discovery of G-quadruplex forming sequences and their functional relevance in plants

Rohini Garg*, Jyoti Aggarwal, Bijal Thakkar

National Institute of Plant Genome Research (NIPGR), Aruna Asaf Ali Marg, New Delhi, India

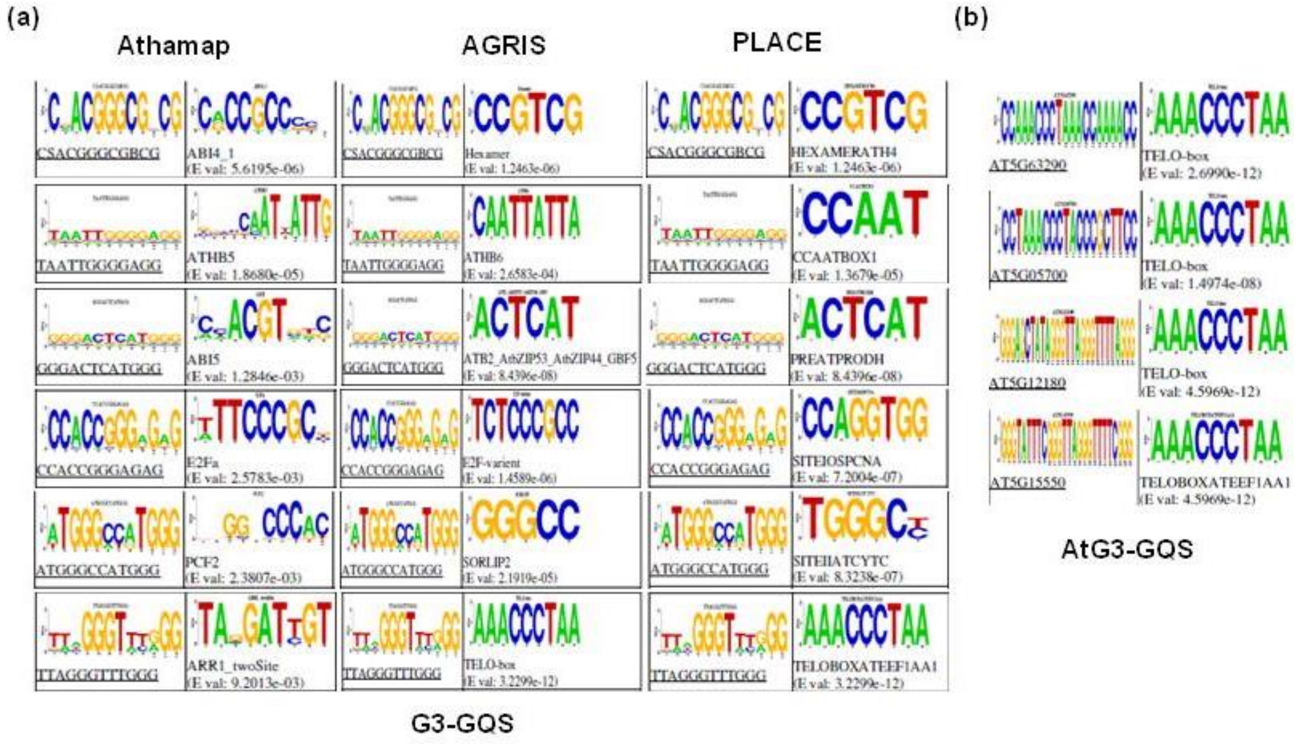

Figure S1. a) Enrichment of transcription factor (TF) binding sites in G3-GQS (G3L1-7, G3L1-3) identified in 1 kb promoters of all plant species as predicted by Athamap, AGRIS and PLACE databases. (b) TF-binding sites identified in Arabidopsis G3-GQS motifs.

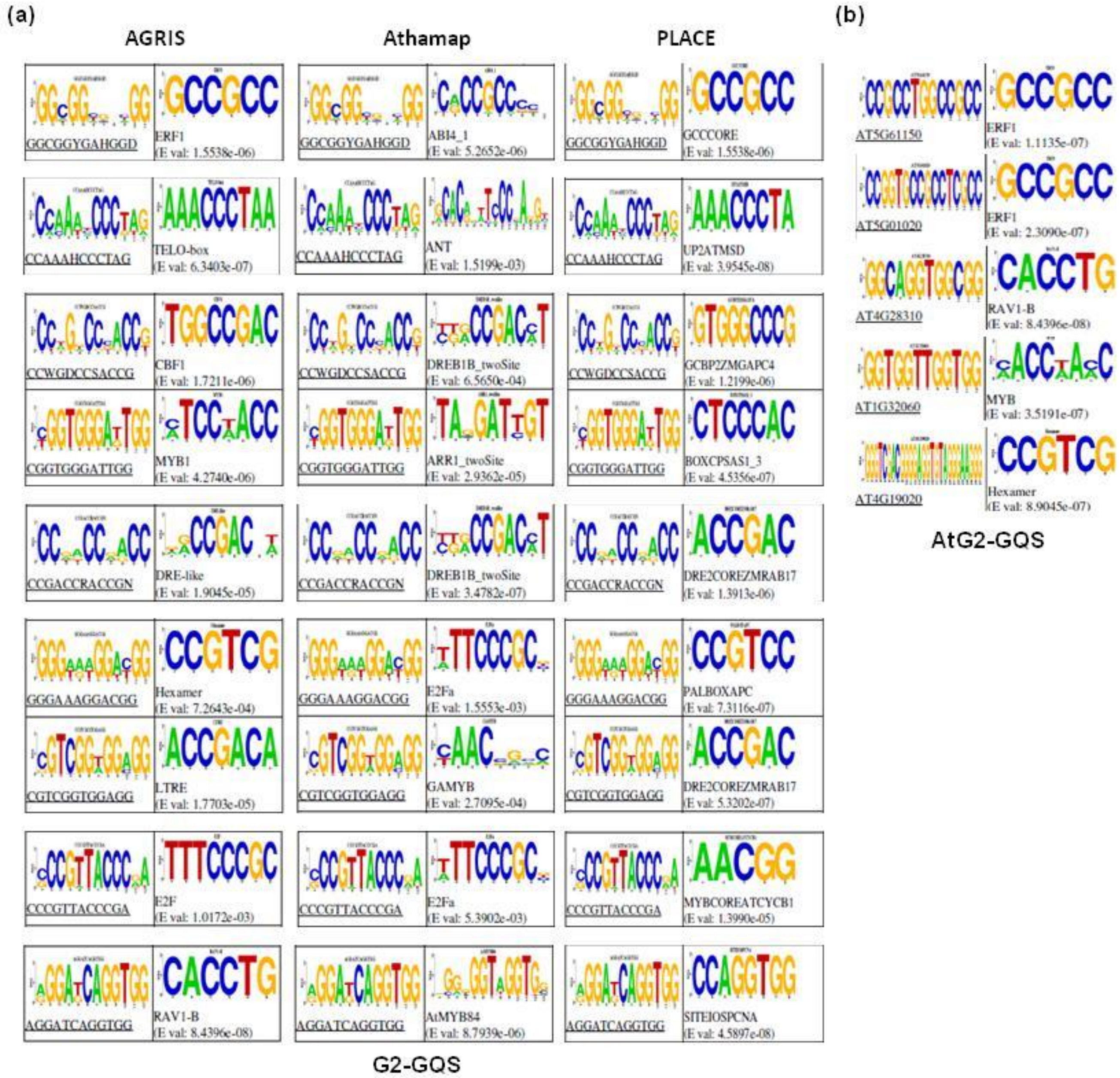

Figure S2. a) Enrichment of transcription factor (TF) binding sites in G2-GQS (G2L1-4, G2L1-2 and G2Ll) identified in 1 kb promoters of all plant species as predicted by AGRIS, Athamap and PLACE databases. (b) TF-binding sites identified in Arabidopsis G2-GQS motifs.

Table S1. Number of GQSes in various genomic features of different plant species.

A. thaliana	gene	mRNA	exon	CDS	3´UTR	5´UTR	Promoter	Intergenic	Intron
G2L1	2925	2893	2811	2465	51	65	529	4593	114
G2L1-2	4886	4836	4642	3872	98	103	876	6775	244
G2L1-4	19564	19381	18364	14627	482	343	3354	22656	1200
G3L1-3	47	45	40	25	5	2	32	213	7
G3L1-7	219	214	195	142	11	9	94	1000	24
L. japonicus									
G2L1	3048	3048	2115	2115	ND	ND	1413	28090	933
G2L1-2	5139	5139	3631	3631	ND	ND	2343	45229	1508
G2L1-4	16392	16392	12567	12567	ND	ND	6133	124511	3825
G3L1-3	279	279	48	48	ND	ND	184	3245	231
G3L1-7	671	671	199	199	ND	ND	500	8235	472
M. truncatula									
G2L1	4197	4197	3169	3025	ND	ND	913	16558	1028
G2L1-2	8133	8133	5735	5396	ND	ND	1873	32681	2398
G2L1-4	30010	30005	21641	20381	ND	ND	6021	105887	8369
G3L1-3	545	545	150	140	ND	ND	232	3445	395
G3L1-7	1143	1142	429	399	ND	ND	482	6963	714
B. rapa									
G2L1	5126	5124	4944	4670	121	153	812	10184	182
G2L1-2	8105	8102	7703	7217	213	273	1355	17407	402
G2L1-4	29434	29425	27239	25707	759	773	5080	66351	2195
G3L1-3	78	78	62	41	10	11	60	580	16
G3L1-7	353	352	300	256	22	22	164	2432	53
G. max									
G2L1	9899	9898	5933	4981	522	430	1116	54978	3966
G2L1-2	17970	17964	10866	8988	1066	812	2035	94735	7104
G2L1-4	61242	61209	41478	35540	3171	2767	6309	329698	19764
G3L1-3	1627	1627	316	177	92	47	254	9134	1311
G3L1-7	2860	2858	969	689	166	114	457	17329	1891
B.distachyon									
G2L1	16418	16418	12601	12043	157	401	4195	72771	3817
G2L1-2	28134	28134	20712	19542	482	690	8024	137413	7422
G2L1-4	81372	81368	59781	56515	1686	1580	20696	393842	21591
G3L1-3	915	915	244	217	18	9	461	7522	671
G3L1-7	2692	2692	1270	1174	48	48	1232	23352	1422
P. patens									
G2L1	6319	6122	4839	3760	157	922	4810	24904	1480
G2L1-2	12735	12360	9724	7710	390	1624	8455	41499	3011
G2L1-4	46288	45100	36079	30007	1397	4675	20695	120341	10209
G3L1-3	688	656	376	269	14	93	1509	5309	312
G3L1-7	2156	2076	1390	1014	80	296	2863	9982	766
P. vulgaris									
G2L1	4142	4142	3214	2889	132	193	349	27714	928
G2L1-2	7864	7864	5844	5189	291	364	746	58612	2020
G2L1-4	28952	28948	22327	20076	1007	1244	2482	211132	6625
G3L1-3	288	288	176	148	11	17	28	1697	112
G3L1-7	827	827	535	457	33	45	107	7065	292
S. italica									
G2L1	18428	18423	16067	14481	415	1171	7301	130645	2361
G2L1-2	31131	31122	26512	23634	996	1882	13489	249077	4619
G2L1-4	87224	87199	74666	67083	2883	4400	32020	656332	12558
G3L1-3	1024	1024	452	323	41	88	778	11674	572
G3L1-7	2887	2885	1736	1423	91	222	2165	38050	1151
S. bicolor									

G2L1	23505	23501	18416	15092	890	2434	3567	111428	5089
G2L1-2	40095	40083	30288	23655	2151	4482	7330	235076	9807
G2L1-4	113213	113176	84993	67246	6485	11262	19996	749263	28220
G3L1-3	1827	1827	709	326	139	244	572	9882	1118
G3L1-7	4036	4034	1999	1159	278	562	1285	38030	2037
P. trichocarpa									
G2L1	5580	5578	3907	3290	341	276	1014	21754	1673
G2L1-2	10407	10401	7288	6118	702	468	1981	41688	3119
G2L1-4	37761	37728	28926	25371	2195	1360	6133	129252	8835
G3L1-3	608	608	214	111	68	35	187	3393	394
G3L1-7	1346	1345	646	472	109	95	401	7679	700
V.vinifera									
G2L1	7580	7580	1891	1450	244	197	1010	25155	5689
G2L1-2	15531	15531	3762	2904	471	387	2045	52226	11769
G2L1-4	57722	57722	14104	10888	1789	1427	6685	185910	43618
G3L1-3	2004	2004	453	347	60	46	271	6718	1551
G3L1-7	3870	3870	912	697	121	94	573	13574	2958
S. moellendorffii									
G2L1	15707	15707	11624	11134	341	149	2527	20291	4083
G2L1-2	27165	27165	19987	19196	560	231	4123	34817	7178
G2L1-4	100311	100311	73607	70865	1832	910	14159	124538	26704
G3L1-3	1243	1243	850	811	28	11	72	2138	393
G3L1-7	3828	3828	2638	2524	78	36	377	6191	1190
C. arietinum									
G2L1	2135	2132	1648	1569	39	40	219	9352	487
G2L1-2	4557	4545	3277	3123	83	71	553	21007	1280
G2L1-4	20246	20203	15236	14648	344	244	1992	85615	5010
G3L1-3	96	96	48	45	1	2	19	900	48
G3L1-7	349	349	202	182	14	6	84	4207	147
O. sativa									
G2L1	50070	49794	41517	37253	694	3658	8686	135305	8553
G2L1-2	77636	77156	61973	55819	1355	4953	13375	232143	15663
G2L1-4	191466	190324	151072	138301	3849	9353	24317	539252	40394
G3L1-3	2150	2117	1033	706	64	268	1098	11287	1117
G3L1-7	7139	7075	3711	3092	136	499	2870	33620	3428

Table S2. Number of orthologous genes harboring GQSes.

**Dicots**	Genic-G2L1-4	Genic-G3L1-7	1 kb_G2L1-4	1 kb_G3L1-7
A. thaliana	4569	77	1000	35
M. truncatula	2821	3	98	0
B. rapa	3413	21	226	1
G. max	3231	5	96	0
P. vulgaris	3297	5	71	0
C. arietinum	2600	2	48	0
**GQSes in all dicot species**	**1331**	NP	NP	NP
**Monocots**				
O. sativa	14634	2121	5859	639
B. distachyon	13041	353	2808	50
S. italica	11059	380	2580	32
S. bicolor	13620	511	2261	41
**GQSes in all monocot species**	**9715**	**71**	**675**	**3**

NP, Not Present

Table S3. List of orthologous genes harboring GQSes within gene body or promoter region in Arabidopsis and rice.

This table has been provided as separate file in MS Excel format.

Table S4. List of orthologous genes harboring G3-type GQSes within promoter region.

Oryza sativa	Gene description	*Setaria italica*	*Sorghum bicolor*	*Brachypodium distachyon*
LOC_Os01g54390	RNA-binding S4 domain containing protein	Si002389m	Sobic.003G294300	Bradi2g49727
LOC_Os02g58500	PhospholipaseA2 (PLA2)	Si018638m	Sobic.004G357800	Bradi3g60710
LOC_Os08g05540	Conserved protein (DMRT homologue)	Si014452m	Sobic.007G038900	Bradi3g17147

Table S5. List of genes harboring GQSes used for validation for G-quadruplex formation along with predicted TF- binding sites.

		Motifs predicted by STAMP		
**Oligo-id**	**Gene-ID**	**Agris database**	**Athamap database**	**PLACE database**
Os3	LOC_Os12g10100	E2F<br>(E val: 3.0770e-03)	NAM_oneSite<br>(E val: 1.6093e-04)	ANAERO5CONSENSUS<br>(E val: 3.2172e-07)
Os4	LOC_Os01g16610	E2F<br>(E val: 6.1785e-04)	AtMYB84<br>(E val: 1.8830e-07)	ACIIPVPAL2<br>(E val: 4.0457e-13)
Os6	LOC_Os07g03770	E2F-varient<br>(E val: 1.5568e-07)	E2Ff_oneSite<br>(E val: 2.3319e-03)	E2FAT<br>(E val: 2.9025e-06)
Os9	LOC_Os06g04190	E2F-varient<br>(E val: 1.6093e-04)	NAM_oneSite<br>(E val: 1.6093e-04)	IDRSZMFER1<br>(E val: 2.4361e-06)
Os11	LOC_Os02g04430	E2F-varient<br>(E val: 1.6093e-04)	ABI5<br>(E val: 2.4567e-05)	ABRETAEM<br>(E val: 7.1456e-08)
Os5	LOC_Os09g13940	E2F-varient<br>(E val: 1.6093e-04)	ALFIN1<br>(E val: 5.1474e-05)	AMMORESIIUDCRNIA1<br>(E val: 1.3945e-08)
Os7	LOC_Os04g05010	GT<br>(E val: 5.0385e-03)	ALFIN1<br>(E val: 1.7519e-05)	ACIIPVPAL2<br>(E val: 1.7192e-06)
Os10	LOC_Os04g45990	E2F-varient<br>(E val: 1.6093e-04)	AtMYB84<br>(E val: 8.0216e-07)	ACIPVPAL2<br>(E val: 3.3710e-09)
Os13	LOC_Os02g29890	E2F-varient<br>(E val: 1.6093e-04)	NAM_oneSite<br>(E val: 1.6093e-04)	CTRMCAMV35S<br>(E val: 3.2172e-07)
Os18	LOC_Os09g20620	E2F-varient<br>(E val: 1.2123e-04)	AtMYB84<br>(E val: 4.7532e-07)	ACIIPVPAL2<br>(E val: 1.0913e-08)
At3	AT3G05620	E2F<br>(E val: 3.2703e-10)	E2Ff_oneSite<br>(E val: 2.1970e-10)	E2FAT<br>(E val: 2.2581e-10)
At8	AT1G32350	E2F-varient<br>(E val: 1.8404e-05)	E2Fc<br>(E val: 7.2880e-08)	PALBOXAPC<br>(E val: 7.7239e-07)
At12	AT5G06839	MYB1<br>(E val: 5.8386e-08)	GAMYB<br>(E val: 5.6476e-05)	BOXICHS<br>(E val: 8.9373e-14)
At15	AT2G47500	AtMYB2<br>(E val: 7.8391e-09)	AtMYB44_twoSite<br>(E val: 4.9418e-04)	MYBATRD22<br>(E val: 7.8391e-09)
At17	AT5G26940	TGA1<br>(E val: 8.6139e-07)	bZIP911_1<br>(E val: 5.5766e-11)	REGION1OSOSEM<br>(E val: 6.7106e-09)
Ca1	Ca_05841	E2F-varient<br>(E val: 8.5399e-04)	NAM_oneSite<br>(E val: 8.5399e-04)	AGTACSAO<br>(E val: 4.3865e-08)
Ca2	Ca_19695	GT<br>(E val: 7.2494e-07)	AtMYB84<br>(E val: 5.7503e-08)	ACIIPVPAL2<br>(E val: 1.0230e-09)
Gm1	Glyma20g25530	E2F-varient<br>(E val: 8.5399e-04)	NAM_oneSite<br>(E val: 2.6953e-08)	O2F2BE2S1<br>(E val: 3.8951e-06)
Gm2	Glyma05g24940	E2F-varient<br>(E val: 8.5399e-04)	NAM_oneSite<br>(E val: 1.2123e-04)	AMMORESIIUDCRNIA1<br>(E val: 1.7833e-05)
Gm4	Glyma09g29900	E2F-varient<br>(E val: 1.6093e-04)	AtMYB84<br>(E val: 1.4559e-05)	ACIPVPAL2<br>(E val: 3.3710e-09)

Table S6. Databases used for downloading genomes of different plant species.

Organism name	webpages
L. japonicus	ftp://ftp.kazusa.or.jp/pub/lotus/lotus_r2.5/
M. truncatula	http://phytozome.jgi.doe.gov/pz/portal.html#!info?alias=Org_Mtruncatula
P. vulgaris	http://genome.jgi.doe.gov/pages/dynamicOrganismDownload.jsf?organism=Pvulgaris
C. arietinum	http://nipgr.res.in/CGAP/download/genome_sequencing/genome_sequence/ C.arietinum_ICC4958_Draft1.fasta
G. max	http://genome.jgi.doe.gov/pages/dynamicOrganismDownload.jsf?organism=Gmax
A. thaliana	ftp://ftp.arabidopsis.org/home/tair/Sequences/whole_chromosomes/
B. rapa	http://genome.jgi.doe.gov/pages/dynamicOrganismDownload.jsf?organism=BrapaFPsc
S. bicolor	http://genome.jgi.doe.gov/pages/dynamicOrganismDownload.jsf?organism=Sbicolor
S. italica	http://genome.jgi.doe.gov/pages/dynamicOrganismDownload.jsf?organism=Sitalica
O. sativa	MSUv7.0; http://rice.plantbiology.msu.edu
B. distachyon	http://genome.jgi.doe.gov/pages/dynamicOrganismDownload.jsf?organism=Bdistachyon
P. patens	http://genome.jgi.doe.gov/pages/dynamicOrganismDownload.jsf?organism=Ppatens
S. moellendorffii	http://genome.jgi.doe.gov/pages/dynamicOrganismDownload.jsf?organism=Smoellendorffii
V. vinifera	http://genome.jgi.doe.gov/pages/dynamicOrganismDownload.jsf?organism=Vvinifera
P. trichocarpa	http://genome.jgi.doe.gov/pages/dynamicOrganismDownload.jsf?organism=Ptrichocarpa
